# Factors impacting quality of life in multiple system atrophy

**DOI:** 10.3389/fneur.2023.1111605

**Published:** 2023-03-10

**Authors:** Nabila Ali, Vanessa Nesspor, Jee Bang, Sonja W. Scholz, Alexander Pantelyat

**Affiliations:** ^1^Department of Neurology, Johns Hopkins University School of Medicine, Baltimore, MD, United States; ^2^Neurodegenerative Diseases Research Unit, National Institute of Neurological Disorders and Stroke, National Institutes of Health, Bethesda, MD, United States

**Keywords:** multiple system atrophy, quality of life, atypical parkinsonian disorders, activities of daily living, hygiene

## Abstract

**Background:**

Multiple system atrophy (MSA) is an atypical parkinsonian disorder marked by autonomic dysfunction, parkinsonism, cerebellar dysfunction, and poor response to dopaminergic medications such as levodopa. Patient-reported quality of life is an important benchmark for clinicians and clinical trials. The Unified Multiple System Atrophy Rating Scale (UMSARS) allows healthcare providers to rate and assess MSA progression. The MSA-QoL questionnaire is a health-related quality of life scale intended to provide patient-reported outcome measures. In this article, we investigated inter-scale correlations between the MSA-QoL and UMSARS to determine factors impacting the quality of life of patients with MSA.

**Methods:**

Twenty patients at the Johns Hopkins Atypical Parkinsonism Center's Multidisciplinary Clinic with a diagnosis of clinically probable MSA and who filled out the MSA-QoL and UMSARS questionnaires within 2 weeks of each other were included. Inter-scale correlations between MSA-QoL and UMSARS responses were examined. Linear regressions were also performed to examine relationships between both scales.

**Results:**

Significant inter-scale correlations were found between the MSA-QoL and UMSARS, both between MSA-QoL total score and UMSARS Part I subtotal scores and for individual scale items. There were no significant correlations between MSA-QoL life satisfaction rating and UMSARS subtotal scores or any specific UMSARS items. Linear regression analysis found significant associations between MSA-QoL total score and UMSARS Part I and total scores, and between MSA-QoL life satisfaction rating and UMSARS Part I, Part II, and total scores (after adjustment for age).

**Conclusions:**

Our study demonstrates significant inter-scale correlations between MSA-QoL and UMSARS, particularly relating to activities of daily living and hygiene. MSA-QoL total score and UMSARS Part I subtotal scores, which assess patients' functional status, were significantly correlated. The lack of significant associations between MSA-QoL life satisfaction rating and any UMSARS item suggests there may be aspects to quality of life that are not fully captured by this assessment. Larger cross-sectional and longitudinal analyses utilizing UMSARS and MSA-QoL are warranted and modification of the UMSARS should be considered.

## 1. Introduction

Multiple system atrophy (MSA) is an atypical parkinsonian disorder marked by autonomic dysfunction, parkinsonism, cerebellar abnormalities, and poor response to dopaminergic medications such as levodopa (1). The course of MSA is usually aggressive in comparison with that of Parkinson's disease, with a median time to wheelchair confinement of 5 years from disease onset and median time to death of about 10 years from disease onset (2, 3). Clinical manifestations of MSA, including but not limited to motor symptoms, dysphagia, orthostatic hypotension, bladder dysfunction, disordered sleep, and obstructive sleep apnea, significantly impact patient quality of life (4). Factors associated with a more aggressive disease course include early falls, older age at disease onset, severe autonomic failure, and severe urinary retention (5–8).

Clinically, MSA is divided into three subtypes, based on symptom predominance: parkinsonian (MSA-P), cerebellar (MSA-C), or mixed (MSA-mixed). MSA-P presents with predominantly parkinsonian signs such as bradykinesia, rigidity, and tremor; MSA-C with symptoms such as gait and limb ataxia or oculomotor dysfunction; and MSA-mixed with features of both (1). Though several prior studies found shorter survival time and lower health-related quality of life in MSA-P compared to MSA-C, this was not found to be the case in a large multi-center North American MSA cohort (2, 3, 9, 10). There are currently no approved disease-modifying MSA treatments, and multiple clinical trials have failed to improve outcome measures, raising the possibility that outcome measures in MSA should be reconsidered. To optimize clinical trial outcomes and to track disease progression in the clinic, it is important to determine the specific patient-reported disease aspects that affect quality of life.

Health-related quality of life (health-related QoL), as well as survival, is significantly impaired in those with atypical parkinsonian disorders, including MSA (11). Some specific features of MSA, such as autonomic symptoms (e.g., orthostatic hypotension) and cognitive impairment, have already been demonstrated to be associated with more rapid disease progression and poorer quality of life (12–14). One prospective cohort study found that nearly one-third of those with MSA initially presented with symptoms of autonomic dysfunction, and 18.2% initially presented with urinary symptoms specifically (15, 16). MSA is marked by notable lower urinary tract symptoms, which can require intermittent catheterization and is “a major cause of hospitalization and dependence upon carers” (17). Autonomic symptoms such as earlier falls, bladder symptoms, earlier time to bladder catheterization, and severity of autonomic failure have been associated with poorer survival (8). Early stridor onset has also been associated with unfavorable survival (18). Impaired sleep quality is also an important facet of multiple system atrophy. REM sleep behavior disorder may be an early sign of disease onset, and, in such patients, may be associated with increased autonomic dysfunction, more rapid progression of disease, and even a higher risk of death (19).

It is important to understand factors that impact quality of life in individuals with MSA, as quality of life is a potentially modifiable outcome. For this reason, health-related QoL can serve as a benchmark in assessing disease progression and as a potential clinical trial outcome. To fulfill this purpose, valid and reliable methods of quantifying health-related QoL and MSA symptom progression are needed.

The MSA-QoL questionnaire was developed as a health-related QoL scale intended to provide patient-reported outcome measures for this debilitating condition (20). This scale is the first published and validated patient-reported health-related QoL measure for patients with MSA. It contains items addressing motor symptoms; nonmotor symptoms, such as autonomic dysfunction, sexual impairment, and bowel/bladder dysfunction; and emotional/social impacts of MSA, such as feelings of isolation and anxiety about the future (20). Patients and/or caregivers are instructed to rate MSA symptoms over a period of 4 weeks, reporting “no problem,” “slight problem,” “moderate problem,” “marked problem,” or “extreme problem” for each symptom in question, with a final item asking patients to report overall life satisfaction from 0 to 100 by marking a grid.

The Unified Multiple System Atrophy Rating Scale (UMSARS) was developed to objectively rate and assess MSA progression (21). It is commonly used as a primary clinical trial outcome in this condition. UMSARS is composed of four parts: a historical domain that assesses motor and autonomic disability based on clinician scoring with patient and/or care partner input (Part I), a motor examination domain (Part II), an autonomic examination to assess for orthostatic hypotension (Part III), and a final item rating global disability (Part IV) (21). Clinicians are asked to rate patient functional status during the preceding 2 weeks for various items from a scale of 0 to 4, with 0 corresponding to normal/unimpaired function and 4 corresponding to severe impairment. The clinician also assigns a global disability rating from 1 to 5, with a rating of 1 corresponding to complete independence and a rating of 5 to a totally dependent and bedridden state.

The aim of this cross-sectional study specifically is to explore inter-scale correlations between the MSA-QoL items and subscales and UMSARS items and subscales. We investigated areas of concordance and discordance between patient-reported and clinician-scored responses, providing insight into factors affecting quality of life in individuals with MSA. Previous work by Meissner et al. (22) found that MSA-QoL scores were less indicative of disease progression over time than UMSARS scores. Expanding upon this work, this analysis also explores which individual patient-reported outcomes correlated with clinician-scored disease severity, impact, and functional status.

## 2. Materials and methods

Twenty patients who were assessed at the Johns Hopkins Atypical Parkinsonism Center's Multidisciplinary Clinic between 2015 and 2022 by one of the study co-authors (JB, SS, or AP) were included in this study. This study protocol was approved by the Institutional Review Board at Johns Hopkins School of Medicine (Johns Hopkins IRB-2, study number IRB00062534). All participants provided written informed consent for this study. Study inclusion criteria were: (1) clinically probable MSA diagnosis (retrospectively determined according to the recently published MSA diagnostic criteria; “MSA-mixed” designation was assigned when patients had features of both parkinsonism and cerebellar dysfunction without clear predominance of one over the other) and (2) availability of MSA-QoL and UMSARS questionnaires completed within 2 weeks of each other (1). The UMSARS questionnaire was administered during the clinic visit, and the MSA-QoL questionnaire was mailed (or emailed, as per patient preference) to patients to complete and return.

### 2.1. MSA-QoL questionnaire

The MSA-QoL is a 40-item questionnaire that asks patients to rate their level of difficulty with specific mental or physical domains, as well as a question asking patients to mark overall life satisfaction on a scale from 0 to 100. The questionnaire was developed from physician and patient reports and psychometric data analysis and has been demonstrated to be reliable and valid (20). Items include questions about mobility, coordination, ability to complete self-care, other activities of daily living, symptoms of dysautonomia, sleep quality, cognitive function, and social/emotional impacts of the disease. In total, there are 14 motor items, 12 nonmotor items, and 14 emotional/social items. Higher total scores on the MSA-QoL indicate higher levels of impairment, and higher scores on the life satisfaction item correspond with higher life satisfaction.

### 2.2. UMSARS questionnaire

UMSARS is a clinician-scored rating scale to assess the severity of symptoms for patients with MSA. There is a historical component with 12 items (Part I), a motor examination component with 14 items (Part II), an optional autonomic examination component (Part III), and a global disability scale (Part IV). UMSARS has also been demonstrated to be reliable and valid (21). In the UMSARS, symptoms are rated by clinicians over a period of 2 weeks. Higher UMSARS scores indicate greater levels of impairment.

### 2.3. Statistical analysis

The Kruskal–Wallis rank-sum test or Pearson's χ^2^ test was used to compare patient characteristics between groups. Spearman correlations with Benjamini-Hochberg correction (to adjust for multiple comparisons) between MSA-QoL items and UMSARS items were calculated to examine inter-scale associations (23). Linear regression models using MSA-QoL scores as the outcome variable and UMSARS scores as the predictor variable, adjusting for age, gender, disease duration, and disease type, were used to examine the relationships between both scales. Correlation coefficients with *p* < 0.05 were considered to be statistically significant. All statistical analyses were performed in RStudio version 2021.09.2+382 (RStudio, Public Benefit Corporation, 2022).

## 3. Results

Patient characteristics are summarized in Table 1. There were no significant differences in age, sex, or disease duration among MSA types (MSA-C, MSA-P, or MSA-mixed). There were also no significant differences in MSA-QoL total score, MSA-QoL satisfaction rating, UMSARS Part I subtotal score, UMSARS Part II subtotal score, or UMSARS global disability score between MSA types.

**Table 1:** Patient characteristics.

**Characteristic**	**All patients**	**MSA-C**	**MSA-P**	**MSA-mixed**	**Adjusted *p*-value**
*N*	20	5	12	3	
Age in years, mean (SD)	61.3 (7.1)	61.8 (7.3)	61.8 (8.0)	58.0 (3.5)	0.4307
**Sex**, ***n*** **(%)**					
Female	10 (50%)	3 (60%)	6 (50%)	1 (33%)	0.7659
Male	10 (50%)	2 (40%)	6 (50%)	2 (67%)	
Disease duration in years, mean (SD)	5.3 (3.1)	4.6 (2.7)	5.3 (2.9)	6.3 (4.9)	0.8270
**MSA-QoL**					
Total, mean (SD)	86.6 (29.9)	69.0 (29.8)	93.1 (30.3)	92.0 (25.2)	0.3058
Satisfaction, mean (SD)	49% (23)	62% (16)	48% (24)	29% (19)	0.0868
**UMSARS**					
Part I subtotal score, mean (SD)	27.8 (7.8)	24.6 (5.3)	27.9 (9.0)	32.3 (4.2)	0.2089
Part II subtotal score, mean (SD)	30.2 (10.1)	27.4 (11.7)	30.3 (10.7)	34.7 (2.5)	0.5506
Total score (SD)	58.0 (17.0)	52.0 (15.7)	58.2 (19.0)	67.0 (6.1)	0.3446
Global disability score, mean (SD)	3.3 (1.1)	2.8 (1.3)	3.3 (1.1)	4.0 (0.0)	0.2900

**Figure 1:**
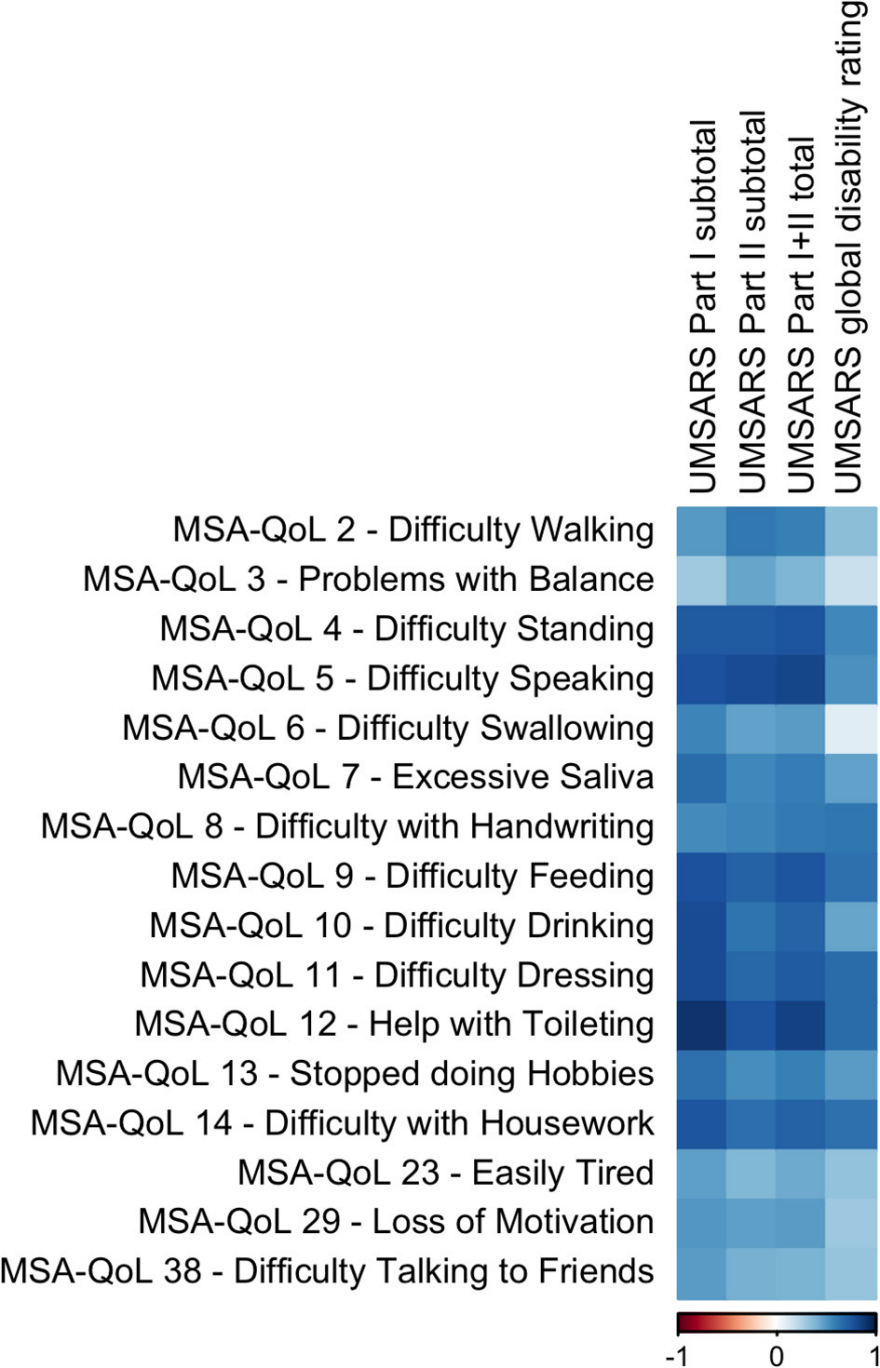
A visual representation of the correlations between UMSARS domains and selected individual MSA-QoL items. A dark blue square represents a correlation coefficient that is close to 1 and a dark red square represents a correlation coefficient that is close to −1, as shown in the legend.

We discovered significant inter-scale correlations between subtotal scores on individual MSA-QoL items and UMSARS Part I (History), UMSARS Part II (Motor Examination), UMSARS total score, UMSARS global disability rating; all *p*-values reported below are adjusted for multiple comparisons. The findings are described in Figure 1, Table 2, and in the text below.

**Table 2:** Correlations of UMSARS section scores with individual MSA-QoL items.

**UMSARS section score**	**MSA-QoL item**	**ρ (adjusted *p*-value)**
UMSARS Part I Score (history)	MSA-QoL 4—Difficulty Standing	0.7519 (**0.0031**)
	MSA-QoL 5—Difficulty Speaking	0.7974 (**0.0014**)
	MSA-QoL 7—Excessive Saliva	0.6705 (**0.0154**)
	MSA-QoL 9—Difficulty Feeding	0.7998 (**0.0010**)
	MSA-QoL 10—Difficulty Drinking	0.8132 (**0.0008**)
	MSA-QoL 11—Difficulty Dressing	0.8176 (**0.0007**)
	MSA-QoL 12—Help with Toileting	0.9164 (< **0.0001**)
	MSA-QoL 13—Stopped doing Hobbies	0.6513 (**0.0203**)
	MSA-QoL 14—Difficulty with Housework	0.7728 (**0.0018**)
	MSA-QoL 2—Difficulty Walking	0.5019 (0.1165)
	MSA-QoL 6—Difficulty Swallowing	0.5791 (0.0530)
	MSA-QoL 8—Difficulty with Handwriting	0.5570 (0.0674)
	MSA-QoL 23—Easily Tired	0.4827 (0.1360)
	MSA-QoL 29—Loss of Motivation	0.5181 (0.1000)
	MSA-QoL 38—Difficulty talking to Friends	0.4922 (0.1263)
UMSARS Part II Score (motor examination)	MSA-QoL 2—Difficulty Walking	0.6291 (**0.0278**)
	MSA-QoL 4—Difficulty Standing	0.7561 (**0.0028**)
	MSA-QoL 5—Difficulty Speaking	0.8172 (**0.0009**)
	MSA-QoL 9—Difficulty Feeding	0.7300 (**0.0052**)
	MSA-QoL 10—Difficulty Drinking	0.6459 (**0.0216**)
	MSA-QoL 11—Difficulty Dressing	0.6926 (**0.0104**)
	MSA-QoL 12—Help with Toileting	0.7814 (**0.0015**)
	MSA-QoL 14—Difficulty with Housework	0.6672 (**0.0162**)
	MSA-QoL 3—Problems with Balance	0.4672 (0.1509)
	MSA-QoL 6—Difficulty Swallowing	0.4711 (0.1474)
	MSA-QoL 7—Excessive Saliva	0.5623 (0.0633)
	MSA-QoL 8—Difficulty with Handwriting	0.5794 (0.0530)
	MSA-QoL 13—Stopped doing Hobbies	0.5423 (0.0792)
	MSA-QoL 29—Loss of Motivation	0.4852 (0.1338)
UMSARS Part I + II Score	MSA-QoL 2—Difficulty Walking	0.5911 (**0.0459**)
	MSA-QoL 4—Difficulty Standing	0.7725 (**0.0018**)
	MSA-QoL 5—Difficulty Speaking	0.8357 (**0.0006**)
	MSA-QoL 7—Excessive Saliva	0.6011 (**0.0410**)
	MSA-QoL 8—Difficulty with Handwriting	0.6116 (**0.0357**)
	MSA-QoL 9—Difficulty Feeding	0.7715 (**0.0018**)
	MSA-QoL 10—Difficulty Drinking	0.7100 (**0.0077**)
	MSA-QoL 11—Difficulty Dressing	0.7519 (**0.0031**)
	MSA-QoL 12—Help with Toileting	0.8529 (**0.0002**)
	MSA-QoL 13—Stopped doing Hobbies	0.5904 (**0.0463**)
	MSA-QoL 14—Difficulty with Housework	0.7298 (**0.0052**)
	MSA-QoL 6—Difficulty Swallowing	0.4953 (0.1233)
	MSA-QoL 23—Easily Tired	0.4467 (0.1772)
	MSA-QoL 29—Loss of Motivation	0.4913 (0.1274)
UMSARS global disability score	MSA-QoL 8—Difficulty with Handwriting	0.6302 (**0.0272**)
	MSA-QoL 9—Difficulty Feeding	0.6600 (**0.0179**)
	MSA-QoL 11—Difficulty Dressing	0.6723 (**0.0149**)
	MSA-QoL 12—Help with Toileting	0.6740 (**0.0148**)
	MSA-QoL 14—Difficulty with Housework	0.6583 (**0.0184**)
	MSA-QoL 4—Difficulty Standing	0.5605 (0.0646)
	MSA-QoL 5—Difficulty Speaking	0.5343 (0.0975)
	MSA-QoL 7—Excessive Saliva	0.5636 (0.1416)
	MSA-QoL 10—Difficulty Drinking	0.4678 (0.1503)
	MSA-QoL 13—Stopped doing Hobbies	0.4910 (0.1275)

Significant correlations (p < 0.05) after adjusting for multiple comparisons are bolded. All other items had significant unadjusted p-values, which were no longer significant after adjustment for multiple comparisons.

Specifically, UMSARS Part I subtotal scores were significantly correlated with MSA-QoL items 4—Difficulty Standing (ρ = 0.7519, adjusted *p* = 0.0031), 5—Difficulty Speaking (ρ = 0.7974, *p* = 0.0014), 7—Excessive Saliva (ρ = 0.6705, *p* = 0.0154), 9—Difficulty Feeding (ρ = 0.7998, *p* = 0.0010), 10—Difficulty Drinking (ρ = 0.8132, *p* = 0.0008), 11—Difficulty Dressing (ρ = 0.8176, *p* = 0.0007), 12—Help with Toileting (ρ = 0.9164, *p* = < 0.0001), 13—Stopped doing Hobbies (ρ = 0.6513, *p* = 0.0203), and 14—Difficulty with Housework (ρ = 0.7728, *p* = 0.0018). UMSARS Part I subtotal scores were also correlated with MSA-QoL items 2—Difficulty Walking (ρ = 0.5019, *p* = 0.1165), 6—Difficulty Swallowing (ρ = 0.5791, *p* = 0.0530), 23—Easily Tired (ρ = 0.4827, *p* = 0.1360), 29—Loss of Motivation (ρ = 0.5181, *p* = 0.1000), and 38—Difficulty Talking to Friends (ρ = 0.4922, *p* = 0.1263), but these correlations were not significant after adjustment for multiple comparisons.

UMSARS Part II subtotal scores were significantly correlated with MSA-QoL items 2—Difficulty Walking (ρ = 0.6291, *p* = 0.0278), 4—Difficulty Standing (ρ = 0.7561, *p* = 0.0028), 5—Difficulty Speaking (ρ = 0.8172, *p* = 0.0009), 9—Difficulty Feeding (ρ = 0.7300, *p* = 0.0052), 10—Difficulty Drinking (ρ = 0.6459, *p* = 0.0216), 11—Difficulty Dressing (ρ = 0.6926, *p* = 0.0104), 12—Help with Toileting (ρ = 0.7814, *p* = 0.0015), and 14—Difficulty with Housework (ρ = 0.6672, *p* = 0.0162). UMSARS Part II subtotal scores were also correlated with MSA-QoL items 3—Problems with Balance (ρ = 0.4672, *p* 0.1509), 6—Difficulty Swallowing (ρ = 0.4711, *p* = 0.1474), 7—Excessive Saliva (ρ = 0.5623, *p* = 0.0633), 8—Difficulty with Handwriting (ρ = 0.5794, *p* = 0.0530), 13—Stopped doing Hobbies (ρ = 0.5423, *p* = 0.0792), and 29—Loss of Motivation (ρ = 0.4852, *p* = 0.1338), but these correlations were not significant after adjustment for multiple comparisons.

UMSARS combined Part I and Part II scores were significantly correlated with MSA-QoL items 2—Difficulty Walking (ρ = 0.5911, *p* = 0.0459), 4—Difficulty Standing (ρ = 0.7725, *p* = 0.0018), 5—Difficulty Speaking (ρ = 0.8357, *p* = 0.0006), 7—Excessive Saliva (ρ = 0.6011, *p* = 0.0410), 8—Difficulty with Handwriting (ρ = 0.6116, *p* = 0.0410), 9—Difficulty Feeding (ρ = 0.7715, *p* = 0.0018), 10—Difficulty Drinking (ρ = 0.7100, *p* = 0.0077), 11—Difficulty Dressing (ρ = 0.7519, *p* = 0.0031), 12—Help with Toileting (ρ = 0.8529, *p* = 0.0002), 13—Stopped doing Hobbies (ρ = 0.5904, *p* = 0.0463), and 14—Difficulty with Housework (ρ = 0.7298, *p* = 0.0052). UMSARS combined Part I and Part II scores were also correlated with MSA-QoL items 6—Difficulty Swallowing (ρ = 0.4953, *p* = 0.1233), 23—Easily Tired (ρ = 0.4467, *p* = 0.1772), and 29—Loss of Motivation (ρ = 0.4913, *p* = 0.1274), but these correlations were not significant after adjustment for multiple comparisons.

UMSARS global disability scores were significantly correlated with individual MSA-QoL items 8—Difficulty with Handwriting (ρ = 0.6302, *p* = 0.0272), 9—Difficulty Feeding (ρ = 0.6600, *p* = 0.0179), 11—Difficulty Dressing (ρ = 0.6723, *p* = 0.0149), 12—Help with Toileting (ρ = 0.6740, *p* = 0.0148), and 14—Difficulty with Housework (ρ = 0.6583, *p* = 0.0184). UMSARS global disability scores were also correlated with MSA-QoL items 4—Difficulty Standing (ρ = 0.5605, *p* = 0.0646), 5—Difficulty Speaking (ρ = 0.5343, *p* = 0.0975), 7—Excessive Saliva (ρ = 0.5636, *p* = 0.1416), 10—Difficulty Drinking (ρ = 0.4678, *p* = 0.1503), and 13—Stopped doing Hobbies (ρ = 0.4910, *p* = 0.1275), but these correlations were not significant after adjustment for multiple comparisons.

Corresponding individual items on UMSARS and MSA-QoL also agreed well with each other, though there were some notable discrepancies. These findings are described in Table 3 and in the text below.

**Table 3:** Correlations of individual UMSARS items with closest corresponding individual MSA-QoL items.

**UMSARS item**	**MSA-QoL item**	**ρ (adjusted *p*-value)**
UMSARS Part I, 7—Walking	MSA-QoL 2—Difficulty Walking	0.6030 (**0.0402**)
UMSARS Part I, 8—Falling	MSA-QoL 3—Problems with Balance	0.2145 (0.5898)
UMSARS Part I, 1—Speech	MSA-QoL 5—Difficulty Speaking	0.7667 (**0.0030**)
UMSARS Part I, 2—Swallowing	MSA-QoL 6—Difficulty Swallowing	0.5244 (0.0939)
UMSARS Part I, 3—Handwriting	MSA-QoL 8—Difficulty with Handwriting	0.5910 (**0.0459**)
UMSARS Part I, 4—Cutting food	MSA-QoL 9—Difficulty Feeding	0.8300 (**0.0005**)
UMSARS Part I, 5—Dressing	MSA-QoL 11—Difficulty Dressing	0.8684 (**0.0001**)
UMSARS Part I, 10—Urinary Function	MSA-QoL 15—Bladder Problems	0.5085 (0.1098)
UMSARS Part I, 12—Bowel Function	MSA-QoL 16—Constipation	0.3467 (0.3334)
UMSARS Part I, 9—Orthostatic Symptoms	MSA-QoL 17—Dizziness when Standing	0.2909 (0.4394)

Significant correlations (p < 0.05) after adjusting for multiple comparisons are bolded.

UMSARS items that correlated significantly with their corresponding MSA-QoL item include UMSARS Walking and MSA-QoL 2—Difficulty Walking (ρ = 0.6030, *p* = 0.0402), UMSARS Speech and MSA-QoL 5—Difficulty Speaking (ρ = 0.7667, *p* = 0.0030), UMSARS Handwriting and MSA-QoL 8—Difficulty with Handwriting (ρ = 0.5910, *p* = 0.0459), UMSARS Cutting Food and MSA-QoL 9—Difficulty Feeding (ρ = 0.8300, *p* = 0.0005), and UMSARS Dressing and MSA-QoL 11—Difficulty Dressing (ρ = 0.8684, *p* = 0.0001). The correlations between UMSARS Swallowing and MSA-QoL 6—Difficulty Swallowing (ρ = 0.5244, *p* = 0.0939) and UMSARS Urinary Function and MSA-QoL 15—Bladder Problems (ρ = 0.5085, *p* = 0.1098) were no longer significant after adjustment for multiple comparisons. There were no significant correlations between UMSARS Falling and MSA-QoL 3—Problems with Balance (ρ = 0.2145, *p* = 0.5898), UMSARS Bowel Function and MSA-QoL 16—Constipation (ρ = 0.3467, *p* = 0.3334), and UMSARS Orthostatic Symptoms and MSA-QoL 17—Dizziness when Standing (ρ = 0.2909, *p* = 0.4394).

MSA-QoL total score was significantly correlated with UMSARS Dressing (ρ = 0.6034, *p* = 0.0444), UMSARS Urinary Function (ρ = 0.6203, *p* = 0.0384), and UMSARS Facial Expression (ρ = 0.6010, *p* = 0.0481). There were significant correlations between MSA-QoL total score and UMSARS Hygiene (ρ = 0.5385, *p* = 0.0936) and UMSARS Leg Agility (ρ = 0.5826, *p* = 0.0593), but these correlations were no longer significant after adjustment for multiple comparisons.

**Figure 2:**
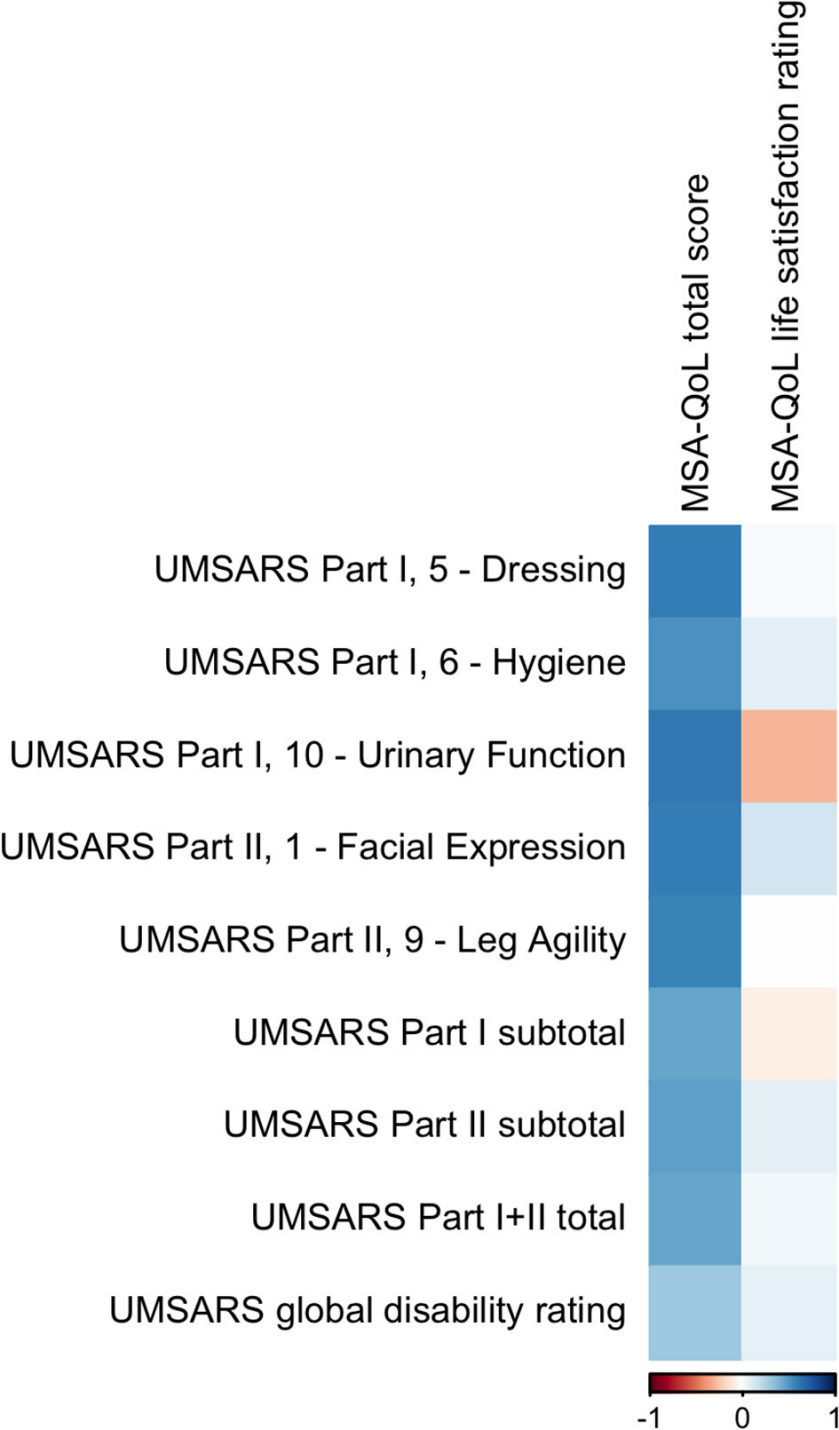
A visual representation of the correlations between MSA-QoL domains and selected individual UMSARS items. A dark blue square represents a correlation coefficient that is close to 1 and a dark red square represents a correlation coefficient that is close to −1, as shown in the legend.

UMSARS Part I subtotal score (ρ = 0.4655, *p* = 0.1695), UMSARS Part II subtotal score (ρ = 0.4817, *p* = 0.1491), and UMSARS total score (obtained from summing UMSARS Part I and Part II subtotal scores) (ρ = 0.4605, *p* = 0.1757) were significantly correlated, but these correlations were no longer significant after adjustment for multiple comparisons. MSA-QoL total score and UMSARS Global Disability score were not significantly correlated (ρ = 0.3106, *p* = 0.4182). These findings are also represented in Figure 2, Table 4.

**Table 4:** Correlations of MSA-QoL scores with UMSARS items.

**MSA-QoL score**	**UMSARS items**	**ρ (adjusted p-value)**
MSA-QoL total score	UMSARS Part I, 5—Dressing	0.6084 (**0.0444**)
	UMSARS Part I, 10—Urinary Function	0.6203 (**0.0384**)
	UMSARS Part II, 1—Facial Expression	0.6010 (**0.0481**)
	UMSARS Part I, 6—Hygiene	0.5385 (0.0936^*^)
	UMSARS Part II, 9—Leg Agility	0.5826 (0.0593^*^)
	UMSARS Part I Total	0.4655 (0.1695^*^)
	UMSARS Part II Total	0.4817 (0.1491^*^)
	UMSARS Part I + Part II Total Score	0.4605 (0.1757^*^)
	UMSARS Global Disability Score	0.3106 (0.4182)
MSA-QoL life satisfaction rating	UMSARS Part I, 11—arising from Chair	−0.6006 (0.0574^*^)
	UMSARS Part I total	−0.0659 (0.9001)
	UMSARS Part II total	0.0996 (0.8335)
	UMSARS Part I + Part II total score	0.0451 (0.9369)
	UMSARS Global Disability Score	0.0821 (0.8677)

Significant correlations (p < 0.05) after adjusting for multiple comparisons are bolded. Asterisked items had significant unadjusted p-values, which were no longer significant after adjustment for multiple comparisons. All other items were not significant.

There was a moderate negative correlation between MSA-QoL life satisfaction rating and UMSARS Arising from Chair (ρ = −0.6006, *p* = 0.0574), but this correlation was not significant after adjustment for multiple comparisons. There was a significant correlation between MSA-QoL life satisfaction and MSA-QoL item 17—Dizziness when Standing (ρ = −0.6017, *p* = 0.0481). There were no other significant correlations between MSA-QoL life satisfaction rating and any other MSA-QoL individual items or any UMSARS individual items or subtotal scores, including UMSARS Part I total (ρ = −0.0659, *p* = 0.9001), UMSARS Part II total (ρ = 0.0996, *p* = 0.8335), UMSARS total score (ρ = 0.0451, *p* = 0.9369), and UMSARS global disability score (ρ = 0.0821, *p* = 0.8677). These findings are also represented in Figure 2.

In an exploratory analysis, we also considered associations between the MSA-QoL and UMSARS questionnaires using linear regression models, as shown in Tables 5, 6. MSA-QoL total scores were not significantly associated with age, sex, disease duration, or MSA type alone (all *p* > 0.32). Parsimony was assessed using the Akaike Information Criterion, which showed that the addition of the covariates age, sex, disease duration, and MSA type did not improve the parsimony of the models (24, 25). MSA-QoL total score was significantly associated with UMSARS Part I scores (*p* = 0.0058 without adjusting for age, sex, disease duration, or MSA type) and UMSARS combined Part I and II scores (*p* = 0.0170) and tended to be associated with UMSARS Part II scores (*p* = 0.0590) and UMSARS global disability rating (*p* = 0.0563).

**Table 5:** Linear regression model results for MSA-QoL total score, not adjusted for age, sex, disease duration, or MSA type.

	**MSA-QoL predictors**			
	**UMSARS Part I**	**UMSARS Part II**	**UMSARS Part I** + **Part II total**	**UMSARS global disability**
Beta (SE)	2.278 (0.723)	1.2784 (0.6317)	0.9292 (0.3513)	12.133 (5.924)
*p*-value	**0.0058**	0.0590	**0.0170**	0.0563

*P* values < 0.05 are in bold.

**Table 6:** Linear regression model results for MSA-QoL life satisfaction rating, with age as a covariate.

	**MSA-QoL predictors**			
	**UMSARS Part I**	**UMSARS Part II**	**UMSARS Part I** + **Part II total**	**UMSARS global disability**
Beta (SE)	−0.004043 (0.006526)	−0.003242 (0.005656)	−0.002019 (0.003203)	−0.010472 (0.046319)
*p*-value	0.5443	0.5744	0.5374	0.8240
*F*-statistic *p*-value	**0.0456**	**0.0468**	**0.0453**	0.0537

*P* values < 0.05 are in bold.

MSA-QoL life satisfaction ratings were significantly associated with age alone (*b* = 0.0182, *p* = 0.0144) and sex alone (*b* = 0.2087, *p* = 0.0463), with female sex associated with higher life satisfaction rating. MSA-QoL life satisfaction ratings were not significantly associated with disease duration or MSA type. After adjustment for age, MSA-QoL life satisfaction was significantly associated with UMSARS Part I scores (*p* = 0.0456 after adjustment for age), UMSARS Part II scores (*p* = 0.0468), and UMSARS combined Part I and II scores (*p* = 0.0453).

## 4. Discussion

This study found inter-scale correlations between UMSARS subscale scores and individual MSA-QoL items. MSA-QoL items relating to activities of daily living, such as feeding oneself, dressing oneself, and toileting, were significantly correlated with UMSARS Part I (History) subtotal scores. MSA-QoL items relating to difficulty standing, speaking, drinking, and handwriting, as well as excessive salivation and difficulty with housework, were also significantly correlated with UMSARS Part I subtotal scores. These correlations ranged from ρ = 0.6513 to 0.9164. Many of these MSA-QoL items were also significantly correlated with UMSARS Part II (Motor Examination) subtotal scores, including: difficulty standing, difficulty speaking, difficulty feeding, difficulty with housework, requiring help with toileting, and difficulty walking. These correlations also ranged from ρ = 0.6291 to 0.8172.

UMSARS total scores, obtained by summing UMSARS Part I and Part II scores, were significantly correlated (ρ = 0.5904–0.8529) with 11 individual MSA-QoL items: difficulty walking, difficulty standing, difficulty speaking, excessive salivation, difficulty with handwriting, difficulty feeding, difficulty drinking, difficulty dressing, requiring help with toileting, stopped doing hobbies, and difficulty with housework. UMSARS global disability ratings were significantly correlated (ρ = 0.6302–0.6740) with five individual MSA-QoL items: difficulty with handwriting, difficulty feeding, difficulty dressing, requiring help with toileting, and difficulty with housework. These findings suggest that disease severity, as assessed by the UMSARS total score and by the clinician assessment of UMSARS global disability, is correlated with the ability to complete activities of daily living and other activities related to independence and dignity, such as toileting independently. Autonomic symptoms, such as excessive salivation, may also be associated with disease severity.

We found no significant correlations between MSA-QoL life satisfaction rating and any UMSARS item, or with any other MSA-QoL item other than 17—Dizziness when Standing (ρ = −0.6017, *p* = 0.0481). This suggests that orthostatic symptoms may be an important correlate of life satisfaction, but also that there may be aspects to patient satisfaction with quality of life that are not fully captured by these assessments and may be determined by external factors. These factors might include degree of care partner support, resilience, and levels of optimism/pessimism. It is important to note that the small sample size of this study may also have limited our ability to detect significant correlations.

MSA-QoL total scores, obtained from summing all patient-reported responses to questionnaire items, were correlated to UMSARS Part I subtotal scores (ρ = 0.4655, *p* = 0.1695), though this correlation was no longer significant after adjustment for multiple comparisons. This is likely because both domains assess patient functional status, albeit in differing ways: the MSA-QoL assesses patient functional status through patient/caregiver self-report, while UMSARS involves clinician interview of the patient/caregiver. Notably, MSA-QoL total score was also significantly associated with the following UMSARS individual items: dressing, urinary function, and facial expression. This suggests that issues related to urinary incontinence (likely impacting patients' sense of dignity as well as hygiene) and independence related to dressing may be correlated with quality of life in those with MSA.

While most individual items on UMSARS that had corresponding MSA-QoL counterparts correlated well (ρ = 0.5910–0.8684) with each other, there were some notable exceptions, especially for the items related to falling, dizziness/orthostatic symptoms, and bowel function/constipation. The lack of expected findings may be due to differences in how these questionnaires are administered and interpreted. To elaborate, the MSA-QoL is filled out by the patient and/or caregiver, whereas UMSARS ratings are determined from clinicians' perceptions of patient responses, the latter adding an additional interpretive layer. Additionally, the MSA-QoL questionnaire also instructs patients to rate symptoms over a period of 4 weeks, whereas UMSARS instructs providers to rate symptoms over a period of 2 weeks. Finally, while certain items on the MSA-QoL questionnaire and the UMSARS may pertain to similar domains, subtle differences in the phrasing of prima facie corresponding inter-scale items may lead to different responses. For example, MSA-QoL item 17—Dizziness when Standing asks patients to rate severity of dizziness while standing up, while the corresponding UMSARS item asks about “orthostatic symptoms” more generally, including dizziness but also syncope, visual disturbances, and neck pain.

Limitations of this study include its small sample size, cross-sectional nature, and lack of autopsy confirmation for MSA diagnosis. The small sample size restricted our ability to detect significant differences in our multivariate linear models. The cross-sectional nature of our study also meant we could not examine whether changes in UMSARS scores could predict changes in MSA-QoL over time. Additionally, the administered questionnaires did not formally assess other comorbidities such as depression, meaning this is an unmeasured factor that may impact quality of life in our cohort.

It is also important to note that, while significant correlations were found between multiple items, correlation does not necessarily imply causation. Any observed associations between variables may reflect true correlations; confounding by disease severity, comorbidities, or other variables; or chance. To reduce the possibility of the last, we have adjusted all correlations for multiple comparisons. Moreover, we emphasize that this is an exploratory analysis, as our small sample size limits the ability to generalize from our findings. Therefore, our findings should be interpreted conservatively. Nonetheless, the reported results provide useful information to inform future larger longitudinal studies.

To summarize, our study observed significant inter-scale correlations between multiple UMSARS items and MSA-QoL items. Particularly, MSA-QoL items relating to activities of daily living, hygiene, independence, and other basic functions were found to have significant correlations with MSA disease severity as determined from UMSARS Part I, Part II, and combined Part I and II scores. This is consistent with other work, which has demonstrated that autonomic symptoms are associated with more rapid disease progression in MSA (12). Clinicians and clinical researchers should consider the importance of these outcomes when assessing quality of life in patients with MSA. In particular, hygiene and dignity issues, especially those related to toileting or to urinary incontinence, may be an important and highly specific benchmark when considering quality of life and symptom severity in patients with MSA, as has already been suggested (17).

These results also suggest the possibility of creating a more focused assessment that may accurately capture important aspects of key symptom severity and impact on quality of life. While the full MSA-QoL questionnaire provides valuable information about patient quality of life, the entire survey may be too burdensome and time-consuming to complete during a time-limited healthcare visit. By focusing on activities of daily living and hygiene, which correlate well to UMSARS items, clinicians may be able to guide history-taking to focus on patient-reported outcomes that correlate with clinician-scored outcomes. We also suggest that there may be aspects to overall quality of life that are not fully captured by the UMSARS and that a revision of this assessment should be considered. Although small and cross-sectional in nature, our study draws clear associations between clinician-administered and patient-reported outcomes relevant to quality of life in MSA.

## Data availability statement

The raw data supporting the conclusions of this article will be made available by the authors, without undue reservation.

## Ethics statement

The studies involving human participants were reviewed and approved by the Institutional Review Board at Johns Hopkins School of Medicine (Johns Hopkins IRB-2, study number IRB00062534). The patients/participants provided their written informed consent to participate in this study.

## Author contributions

NA analyzed the data and wrote the original manuscript. AP conceived of the research idea, verified the analytical methods used, and edited the manuscript. AP, VN, JB, and SS were involved in participant recruitment and data-gathering. All authors have read and edited the manuscript and approved its submission.

## References

[1] Wenning GK Stankovic I Vignatelli L Fanciulli A Calandra-Buonaura G Seppi K. The movement disorder society criteria for the diagnosis of multiple system atrophy. Mov Disord. (2022) 37:1131–48. 10.1002/mds.29005 35445419 PMC9321158

[2] Watanabe H Saito Y Terao S Ando T Kachi T Mukai E. Progression and prognosis in multiple system atrophy: an analysis of 230 Japanese patients. Brain. (2002) 125(Pt 5):1070–83. 10.1093/brain/awf117 11960896

[3] Low PA Reich SG Jankovic J Shults CW Stern MB Novak P. Natural history of multiple system atrophy in the USA: a prospective cohort study. Lancet Neurol. (2015) 14:710–9. 10.1016/S1474-4422(15)00058-7 26025783 PMC4472464

[4] Chelban V Catereniuc D Aftene D Gasnas A Vichayanrat E Iodice V. An update on MSA: premotor and non-motor features open a window of opportunities for early diagnosis and intervention. J Neurol. (2020) 267:2754–70. 10.1007/s00415-020-09881-6 32436100 PMC7419367

[5] Klockgether T Lüdtke R Kramer B Abele M Bürk K Schöls L. The natural history of degenerative ataxia: a retrospective study in 466 patients. Brain. (1998) 121:589–600. 10.1093/brain/121.4.589 9577387

[6] O'Sullivan SS Massey LA Williams DR Silveira-Moriyama L Kempster PA Holton JL. Clinical outcomes of progressive supranuclear palsy and multiple system atrophy. Brain. (2008) 131(Pt 5):1362–72. 10.1093/brain/awn065 18385183

[7] Iodice V Lipp A Ahlskog JE Sandroni P Fealey RD Parisi JE. Autopsy confirmed multiple system atrophy cases: mayo experience and role of autonomic function tests. J Neurol Neurosurg Psychiatry. (2012) 83:453–9. 10.1136/jnnp-2011-301068 22228725 PMC3454474

[8] Coon EA Sletten DM Suarez MD Mandrekar JN Ahlskog JE Bower JH. Clinical features and autonomic testing predict survival in multiple system atrophy. Brain. (2015) 138(Pt 12):3623–31. 10.1093/brain/awv274 26369944 PMC4840547

[9] Schulz JB Klockgether T Petersen D Jauch M Müller-Schauenburg W Spieker S. Multiple system atrophy: natural history, MRI morphology, and dopamine receptor imaging with 123IBZM-SPECT. J Neurol Neurosurg Psychiatry. (1994) 57:1047–56. 10.1136/jnnp.57.9.1047 8089667 PMC1073125

[10] Du JJ Wang T Huang P Cui S Gao C Lin Y. Clinical characteristics and quality of life in Chinese patients with multiple system atrophy. Brain Behav. (2018) 8:e01135. 10.1002/brb3.1135 30378279 PMC6305933

[11] Winter Y Spottke AE Stamelou M Cabanel N Eggert K Höglinger GU. Health-related quality of life in multiple system atrophy and progressive supranuclear palsy. Neurodegener Dis. (2011) 8:438–46. 10.1159/000325829 21576919

[12] Tada M Onodera O Tada M Ozawa T Piao YS Kakita A. Early development of autonomic dysfunction may predict poor prognosis in patients with multiple system atrophy. Arch Neurol. (2007) 64:256–60. 10.1001/archneur.64.2.256 17296842

[13] Zhang L Cao B Ou R Wei QQ Zhao B Yang J. Non-motor symptoms and the quality of life in multiple system atrophy with different subtypes. Parkinsonism Relat Disord. (2017) 35:63–8. 10.1016/j.parkreldis.2016.12.007 27993522

[14] Zhang L Cao B Zou Y Wei QQ Ou R Zhao B. Frontal lobe function, behavioral changes and quality of life in patients with multiple system atrophy. Restor Neurol Neurosci. (2019) 37:11–9. 10.3233/RNN-180862 30741706

[15] Sakakibara R Panicker J Simeoni S Uchiyama T Yamamoto T Tateno F. Bladder dysfunction as the initial presentation of multiple system atrophy: a prospective cohort study. Clin Auton Res. (2019) 29:627–31. 10.1007/s10286-018-0550-y 30043182

[16] Figueroa JJ Singer W Parsaik A Benarroch EE Ahlskog JE Fealey RD. Multiple system atrophy: prognostic indicators of survival. Mov Disord. (2014) 29:1151–7. 10.1002/mds.25927 24909319 PMC4139446

[17] Ogawa T Sakakibara R Kuno S Ishizuka O Kitta T Yoshimura N. Prevalence and treatment of LUTS in patients with Parkinson disease or multiple system atrophy. Nat Rev Urol. (2017) 14:79–89. 10.1038/nrurol.2016.254 27958390

[18] Giannini G Calandra-Buonaura G Mastrolilli F Righini M Bacchi-Reggiani ML Cecere A. Early stridor onset and stridor treatment predict survival in 136 patients with MSA. Neurology. (2016) 87:1375–83. 10.1212/WNL.0000000000003156 27566741

[19] Giannini G Mastrangelo V Provini F Droghini A Cecere A Barletta G. Progression and prognosis in multiple system atrophy presenting with REM behavior disorder. Neurology. (2020) 94:e1828–34. 10.1212/WNL.0000000000009372 32234825

[20] Schrag A Selai C Mathias C Low P Hobart J Brady N. Measuring health-related quality of life in MSA: the MSA-QoL. Movement Disorders. (2007) 22:2332–8. 10.1002/mds.21649 17914730

[21] Wenning GK Tison F Seppi K Sampaio C Diem A Yekhlef F. Development and validation of the Unified Multiple System Atrophy Rating Scale (UMSARS). Mov Disord. (2004) 19:1391–402. 10.1002/mds.20255 15452868

[22] Meissner WG Foubert-Samier A Dupouy S Gerdelat-Mas A Debs R Marquant F. Assessment of quality of life with the multiple system atrophy health-related quality of life scale. Mov Disord. (2012) 27:1574–7. 10.1002/mds.25174 23033055

[23] Benjamini Y Hochberg Y. Controlling the false discovery rate: a practical and powerful approach to multiple testing. J R Stat Soc Series B Stat Methodol. (1995) 57:289–300. 10.1111/j.2517-6161.1995.tb02031.x

[24] Akaike H. Information theory and an extension of the maximum likelihood principle. In:Parzen E Tanabe K Kitagawa G, editors. Selected Papers of Hirotugu Akaike [Internet]. New York, NY: Springer (1998), p. 199–213. (Springer Series in Statistics). 10.1007/978-1-4612-1694-0_15

[25] Akaike H. A new look at the statistical model identification. IEEE Trans Automat Contr. (1974) 19:716–23. 10.1109/TAC.1974.1100705

