# Supplementary material for: Factors impacting quality of life in multiple system atrophy
Source: Front Neurol. 2023 Mar 10;14:1111605. doi: 10.3389/fneur.2023.1111605 (PMC10036583; doi:10.3389/fneur.2023.1111605)
Supplement: Supplementary file 1 [file Table_1.pdf]

Supplementary Information

Table S1. *Corresponding MSA-QoL and UMSARS Part I questionnaire items.*

MSA-QoL Item	Question Text	UMSARS Item	Question Text
2	Had difficulty walking?	Part I, 7	Walking
3	Had problems with your balance?	Part I, 8	Falling (rate the past month)
4	Had difficulty standing up without support?		
5	Had difficulty speaking?	Part I, 1	Speech
6	Had difficulty swallowing food?	Part I, 2	Swallowing
7	Had too much saliva or drooling?		
8	Had difficulty with handwriting?	Part I, 3	Handwriting
9	Had difficulty feeding yourself?	Part I, 4	Cutting food and handling utensils
10	Had difficulty drinking fluids?		
11	Had difficulty dressing yourself?	Part I, 5	Dressing
12	Needed help to go to the toilet?		
13	Had to stop doing things that you liked to do, e.g., your hobbies?		
14	Had difficulty doing things around the house, e.g., housework?		
15	Experienced bladder problems?	Part I, 10	Urinary function
16	Experienced problems with constipation?	Part I, 12	Bowel function
17	Experienced dizziness when standing up?	Part I, 9	Orthostatic symptoms
23	Been feeling tired very quickly (without exerting yourself)?		
38	Had difficulty talking to friends about your illness?		
		Part I, 6	Hygiene
		Part II, 1	Facial Expression
		Part II, 9	Leg Agility
		Part II, 11	Arising from Chair
		Part II, 12	Posture
